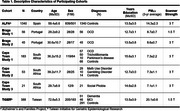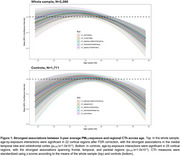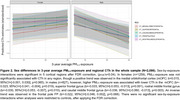# PM2.5 exposure and regional cortical thickness in >2,000 adults from the ENIGMA‐Environment Working Group

**DOI:** 10.1002/alz70860_105317

**Published:** 2025-12-23

**Authors:** Lauren Salminen, Xinhui Wang, Teresa Monreal, Beau MacDonald, John P Wilson, Siyuan Shen, Aaron van Donkelaar, Chi Li, Randall V Martin, Christine Lochner, Kayleigh Beukes, Dan J Stein, Pedro Morgado, Maria Picó Pérez, Jen‐Hau Chen, Yen‐Ching Chen, Natalia Vilor‐Tejedor, Juan Domingo Gispert, Sherezade Fuentes‐Julian, Jordi Huguet, Arcadi Navarro, Paul M. Thompson, Megan M Herting

**Affiliations:** ^1^ Mark and Mary Stevens Neuroimaging and Informatics Institute, Keck School of Medicine, University of Southern California, Marina del Rey, CA, USA; ^2^ Keck School of Medicine, University of Southern California, Los Angeles, CA, USA; ^3^ Spatial Sciences Institute of USC, Los Angeles, CA, USA; ^4^ Washington University in St. Louis, St. Louis, MO, USA; ^5^ Stellenbosch University, Stellenbosch, South Africa; ^6^ University of Minho, Braga, Portugal; ^7^ College of Medicine, National Taiwan University, Taipei, Taiwan; ^8^ Institute of Epidemiology and Preventive Medicine, College of Public Health, National Taiwan University, Taipei, Taiwan; ^9^ BarcelonaBeta Brain Research Center (BBRC), Barcelona, Spain

## Abstract

**Background:**

Exposure to ambient fine particulate matter (<2.5µm; PM2.5) increases risk for suboptimal brain aging and dementia. Here we examined exposure associations with regional cortical thickness (CTh) across seven international datasets within the ENIGMA‐Environment consortium.

**Method:**

Data were collected from 2,086 adults (60% female), ages 18‐89 years (M=57.6±14.1); 1,711 were cognitively unimpaired controls and 375 had diagnoses of mild cognitive impairment (*n* = 46), dementia (*n* = 51), obsessive‐compulsive disorder (*n* = 158), trichotillomania (*n* = 50), methamphetamine use disorder (*n* = 29), social anxiety disorder (*n* = 21), gambling disorder (*n* = 10), or Parkinson's disease (*n* = 10) (Table 1). Satellite‐based estimates of residential PM_2.5_ were quantified as 3‐year average exposures prior to MRI. We tested exposure associations with FreeSurfer‐derived CTh (34 bilateral regions) using linear mixed models, adjusting for age, age^2^, sex, education, race, diagnosis, age‐by‐sex interactions, with random effects of cohort and scanner. Secondarily, we examined PM_2.5_ interactions with age and sex. FDR corrections were applied per analysis.

**Results:**

PM_2.5_ exposure was not associated with CTh in the whole sample but was inversely associated with CTh in the superior temporal gyrus (STG; p_FDR_=0.03) in controls. We observed a significant quadratic PM_2.5_ effect over age across most regions, with negative associations at young and older ages, and positive associations during midlife (Figure 1). In the whole sample only, sex‐by‐exposure interactions were significant in five frontal regions. Exposures were inversely associated with CTh in males, but were not associated with CTh in females (Figure 2). Interaction effects could not be explained by PM_2.5_ exposure differences (t(2084)=‐1.5, *p* = 0.130), and although age differed significantly by sex (t(2084)=‐2.8, *p* = 0.005; male=58.7±14.5, female=56.9±13.8), these differences likely are not meaningful.

**Conclusion:**

PM_2.5_ was inversely associated with CTh in the STG of controls only. Age‐by‐exposure interactions revealed a quadratic effect of PM2.5 on CTh across the lifespan, possibly indicating biphasic exposure‐related neurodegeneration, where CTh increases with early pathology and declines as neurodegeneration progresses. Discordant sex effects may reflect normal sexual dimorphism that is exacerbated by air pollution, but more work is needed to better understand these results.